# Allergic diseases of the skin and drug allergies – 2011. Is the impact of atopic conditions on children and adolescent health related quality of life modified by mental health? Results from a population-based cross-sectional study

**DOI:** 10.1186/1939-4551-6-S1-P98

**Published:** 2013-04-23

**Authors:** Uwe Matterne, Jochen Schmitt, TL Diepgen, CJ Apfelbacher

**Affiliations:** 1 Department of Clinical Social Medicine, Occupational and Environmental Dermatology and Health Systems Research, University Hospital Heidelberg, Germany; 2 Department of Dermatology, University Hospital Carl Gustav Carus, Technical University, Dresden, Germany; 3 Medical Sociology, Institute of Epidemiology and Preventive Medicine, University of Regensburg, Germany, Regensburg, Germany

## Background

Eczema, hay fever and asthma are global health problems. The three conditions have been linked to decreases in health-related-quality-of-life (HRQoL) in adults and children/adolescents. Research also suggests an association of the three conditions with mental health, which in turn is related to HRQoL decreases.

## Methods

The impact of occurrence of the three conditions within the past four weeks on HRQoL was analysed in a population-based sample (N = 6518) of children and adolescents aged 11 – 17. Analyses were adjusted for the other atopic conditions, sociodemographic and clinical variables and stratified for mental health as measured by the Strengths and Difficulties Questionnaire (normal n = 5697, borderline n = 609, abnormal n = 193).

## Results

Eczema (p = 0.015) and hay fever (p = 0.020) within the past four weeks were significantly associated with decreased HRQoL after adjusting for all other variables when no mental health abnormalities were present. No significant associations between these two atopic condition and HRQoL were observed in the groups with borderline or abnormal mental health, respectively. No impact of asthma in any of the groups was observed.

## Conclusions

While the results suggest mental health to have a modifying effect on the relationship between some atopic conditions and HRQoL caution needs to be exercised in interpreting the results as the groups with borderline or abnormal mental health were comparably smaller than the group with normal mental health. In the group with normal mental health small effects were more likely to become significant than in the other two groups.

